# Loneliness and Problematic Media Use: Meta-Analysis of Longitudinal Studies

**DOI:** 10.2196/60410

**Published:** 2025-08-14

**Authors:** Jia Yuin Fam, Niko Männikkö

**Affiliations:** 1 School of Psychology, Faculty of Medical and Life Sciences, Sunway University, 5 Jalan Universiti, Bandar Sunway, 47500, Malaysia, 60 374918622 ext 7183; 2 Centre for Research and Innovation, Oulu University of Applied Sciences, Oulu, Finland

**Keywords:** bidirectional relationship, loneliness, longitudinal studies, meta-analysis, problematic media use

## Abstract

**Background:**

The association between loneliness and problematic media use has been evaluated in longitudinal studies and meta-analyses. However, previous meta-analyses have relied heavily on Pearson correlation coefficients, which may not fully account for the complexities of this association. Therefore, an updated meta-analysis incorporating more robust statistical models is needed.

**Objective:**

This study aimed to examine the longitudinal relationship between loneliness and problematic media use using various statistical models and to explore potential moderators that might influence the strength of this relationship.

**Methods:**

A systematic search was conducted using Scopus, APA PsycArticles, and PubMed to identify eligible studies up to January 24, 2024. Inclusion criteria included studies written in English, published in a peer-reviewed journal, reporting estimates of the longitudinal relationship between loneliness and problematic media use, and a longitudinal study design. Estimates of the longitudinal relationship were synthesized using a random-effects model. The Joanna Briggs Institute Critical Appraisal Checklist was used to assess the risk of bias.

**Results:**

A total of 26 studies involving 24,798 individuals were included in the meta-analysis. Random-effects models revealed bidirectional relationships between loneliness and problematic media use. The longitudinal relationships were weaker when examined using estimated beta coefficients ($r_{LPMU}$=0.10; $r_{PMUL}$=0.09), followed by other statistical models ($r_{LPMU}$=0.10; $r_{PMUL}$=0.10) and the Pearson correlation coefficient ($r_{LPMU}$=0.28; $r_{PMUL}$= 0.29). Subgroup analyses demonstrated stable results for beta coefficients across various study-level characteristics (including country, lag length, measure of problematic media use, and measure of loneliness), except for type of problematic media use (*Q*=16.58; *P*<.001).

**Conclusions:**

This meta-analysis identified smaller effect sizes for the longitudinal relationships between loneliness and problematic media use compared with the previous meta-analysis. The weaker longitudinal relationship observed when using estimated beta coefficients highlights important methodological considerations for future meta-analyses. However, the contour-enhanced funnel plot and Egger regression test revealed an asymmetrical pattern, emphasizing the need for more longitudinal studies in this area.

## Introduction

### Background

The internet has become an integral part of daily life, fundamentally transforming social interaction compared with its predecessors, such as the telegraph, telephone, and television [1]. The internet offers a vast array of opportunities for communication, entertainment, and social connection. However, this ease of access has also brought about concerns regarding problematic media use. Problematic media use involves behaviors that go beyond typical or normative media consumption and may include internet-related activities such as excessive gaming, online shopping, and social network use [2]. Consequently, problematic media use is characterized by an individual’s continued or escalated media or internet over-engagement despite significant impairment to daily functioning across different areas of life [3]. According to Pan et al [4], the pooled average prevalence rate of problematic media use among the general population is around 7%, but it can be significantly higher among youth. In this study, problematic media use includes popular media platforms frequently used by youth, including social media, smartphones, the internet, and gaming. These media platforms have gained considerable attention in research.

### Internet Paradox

The internet paradox [5] suggests that despite the internet’s potential to enhance social connection, it may paradoxically lead to decreased social engagement. While it offers a wider range of social functionalities compared with traditional media, its use for asocial purposes (eg, private entertainment) may hinder face-to-face social interaction [5]. As a result, problematic media use (eg, excessive social media scrolling and compulsive gaming) may lead to unintentional social isolation despite the presence of numerous online connections.

Expanding on this internet paradox, numerous cross-sectional studies have yielded empirical evidence of a link between feelings of loneliness and problematic use of various modern media platforms, including gaming [6], social media [7], smartphones [8], and internet use in general [9]. Collectively, these studies consistently report a significant positive correlation, suggesting that individuals experiencing loneliness tend to engage in higher levels of problematic media use. Further supporting this link, a meta-analysis [10] of 26 studies (mostly cross-sectional) found a positive association between loneliness and problematic media use (pooled *r* range between 0.20 and 0.26). Several reviews have reached similar conclusions about this significant relationship [11-13]. However, a critical limitation identified in these reviews is the predominant use of cross-sectional designs, making it difficult to establish the causal direction in the relationship between loneliness and problematic media use.

### Direction of Causality Between Loneliness and Problematic Media Use

This prompts an inquiry into the directionality of this relationship. Some studies suggest that loneliness may precede problematic media use [14-16], as individuals experiencing loneliness may seek social connection through online platforms. According to the compensatory internet use theory [17], individuals may engage in internet activities to fulfill unmet social needs. The internet offers various avenues for social interaction, ranging from active participation (eg, joining online gaming communities) to passive consumption (eg, browsing stories in social media). Lonely individuals may initially use the internet to satisfy their social needs. However, while this strategy may provide short-term fulfillment, it can lead to a cycle of dependence on the internet to meet these social needs. This dependence can make it difficult for individuals to disengage from the virtual world, potentially resulting in problematic media use.

Conversely, evidence suggests that problematic media use can also contribute to loneliness [18]. Individuals who become excessively reliant on online interactions may neglect face-to-face social connections, potentially leading to feelings of isolation [5]. This aligns with media displacement theory [19], which posits that excessive media consumption can displace real-world social activities. The perceived convenience of the internet, particularly its ability to remove geographical and temporal constraints, might incentivize individuals to substitute face-to-face interactions with online social activities. While successful online relationships exist, research suggests that the quality of online relationships might be poorer than those in physical settings [20]. Online communication is argued to be better suited for maintaining weak ties (superficial connections) rather than strong ties (close relationships).

The contrasting perspectives on the direction of causality between loneliness and problematic media use create a bidirectional relationship, suggesting that the 2 variables may influence each other. However, the strength of the longitudinal relationships appears to vary considerably across studies. While some longitudinal studies support the relationship in both directions, with effect sizes ranging from weak [21] to strong [22], other studies report a nonsignificant longitudinal relationship [23].

In light of this consideration, a meta-analysis that synthesizes past longitudinal findings might provide the best estimate for the longitudinal relationships. Previous meta-analysis examining the longitudinal relationship between loneliness and problematic internet use revealed significant positive correlations in both directions [24]. While their work is valuable, it is noteworthy that they extracted and synthesized Pearson correlation coefficients from past studies (eg, the correlation between loneliness at Time 1 and problematic internet use at Time 2). However, pooling the Pearson coefficients might not be entirely accurate because they do not account for the effect of the outcome variable at Time 1 (ie, problematic internet use at Time 1). For instance, 1 study [25] included in a meta-analysis by Zhang et al [24] reported a Pearson correlation of 0.22 between loneliness at Time 1 and problematic internet use at Time 2. However, their cross-lagged model revealed a nonsignificant standardized beta coefficient of only 0.05 for the same causal relationship. This suggests that the pooled correlations reported by the meta-analysis [24] might be overestimated.

### Objective

The limitations of cross-sectional studies (eg, inability to establish causality), varying past findings from longitudinal studies, and reliance on Pearson correlation coefficients in previous meta-analyses underscore the need for a more robust approach to examining the longitudinal relationships between loneliness and problematic media use. To address these limitations, the current meta-analysis aims to (1) examine the longitudinal relationship between loneliness and problematic media use based on other statistical analyses and (2) explore potential moderators that might influence the strength of the longitudinal relationships.

## Methods

### Overview

The current meta-analysis was conducted following the standards specified in the updated PRISMA (Preferred Reporting Items for Systematic Reviews and Meta-Analyses) 2020 guidelines [26]. This study protocol was preregistered in the Open Science Framework (OSF) Registry on January 17, 2024.

### Search Strategy

A literature search was performed on January 24, 2024, in 3 online databases, namely Scopus, APA PsycArticles, and PubMed. The following search terms were used as keywords: loneliness AND (internet OR “social media” OR “social network” OR gam* OR smartphone) AND (addict* OR disorder OR problem* OR excessive OR patholog*) AND (longitudinal OR prospective). In addition, the gray literature was searched by reviewing the references of included studies.

### Study Inclusion and Exclusion Criteria

The studies were screened in 2 stages by 2 independent researchers. First, duplicate papers were removed, and the remaining papers were screened based on their titles and abstracts to identify potentially relevant studies. In the second stage, the full texts of the shortlisted papers were reviewed for eligibility (Textbox 1).

Textbox 1. **Inclusion and exclusion criteria.**

**Inclusion criteria:**

- Written in English.
- Published in a peer-reviewed journal.
- Reported estimates of the longitudinal relationship between loneliness and problematic media use (eg, reported as correlation coefficient, path coefficient, beta coefficient, odds ratio, or Cohen d; problematic media use included internet gaming disorder, social media disorder, internet addiction, or smartphone addiction).
- A longitudinal study.

**Exclusion criteria:**

- Focused on gambling disorder or gambling-related behavior (eg, loot boxes).
- Did not include measures of loneliness and problematic media use as the investigated variables.
- Were case studies or qualitative studies.
- Focused exclusively on the therapeutic aspects of problematic media use (ie, randomized controlled trial of cognitive behavioral therapy).
- Were preprints, dissertations and theses, conference proceedings and abstracts, or government/industry reports.

### Data Extraction

The following information was extracted from the studies by 2 independent researchers: (1) sample size, (2) study location, (3) age, (4) gender ratio, (5) type of problematic media use recorded, (6) measures of loneliness, (7) measures of problematic media use, (8) number of waves, (9) time lag, and (10) effect size. Any discrepancies during the data extraction process were resolved through discussion.

To investigate the bidirectional relationship between loneliness and problematic media use, 3 effect sizes were recorded whenever possible. First, Pearson correlation coefficients between variables at different time points were documented (eg, the relationship between loneliness at Time 1 and problematic media use at Time 2). If a study reported an effect size other than Pearson *r* (eg, odds ratio), the result was converted into a correlation coefficient using widely accepted formulae [27]. Second, standardized beta coefficients from various statistical models were collected (eg, multiple regression and cross-lagged models). Third, standardized beta coefficients were computed using the following equation [28]:


$$\beta_{Y1.2}=\frac{r_{Y1}-r_{Y2}r_{12}}{1-r_{12}^{2}}$$

To provide further clarity, $\beta_{Y1.2}$ represents the standardized beta coefficient denoting the longitudinal relationship between the independent variable and dependent variable (DV), while controlling for the effect of DV. For instance, it illustrates the effect of loneliness at Time 1 on problematic media use at Time 2, while controlling for the effect of problematic media use at Time 1. Furthermore, $r_{Y1}$ and $r_{Y2}$ denote zero-order correlations between the independent variables and DV. For instance, $r_{Y1}$ signifies the correlation between loneliness at Time 1 and problematic media use at Time 2, whereas $r_{Y2}$ indicates the correlation between problematic media use at Time 1 and Time 2. Finally, $r_{12}$ represents the correlation between loneliness at Time 1 and problematic media use at Time 1.

In a study that included multiple waves of data, the relationships between the 2 closest time points were extracted (eg, Time 1 and Time 2; Time 2 and Time 3).

### Risk of Bias Assessment of the Studies

The included studies were assessed for risk of bias based on the Joanna Briggs Institute (JBI) guidelines [29]. The studies were independently evaluated by 2 authors. The risk of bias assessment for longitudinal studies incorporated 11 domains outlined in the JBI Critical Appraisal Checklist for Cohort Studies. Each domain was assessed as having a high (H; scored as 0), low (L; scored as 1), or unclear (NA; excluded from total score) risk of bias. A high risk of bias indicates that the study’s methodology might significantly affect the measured outcomes. A low risk of bias implies that the methods met quality requirements. An unclear risk definition was used when there was insufficient information to determine the risk of bias. At the end of the assessment, any discrepancies between the 2 researchers were discussed until a consensus was reached.

### Statistical Analysis

The meta-analysis was conducted using the *“*meta” package in the R software (R Foundation for Statistical Computing) environment [30]. We used a random-effects model to compare effect sizes, assuming heterogeneity between samples. Three meta-analyses were conducted separately for the 3 types of effect sizes, accompanied by 95% prediction intervals [31]. Heterogeneity was quantified using the restricted maximum likelihood estimator and I^2^ statistic. I^2^ values between 50% and 75% indicate moderate heterogeneity, while values above 75% reflect high heterogeneity [32].

To delve deeper into the longitudinal relationships between loneliness and problematic media use, subgroup analyses were conducted to identify potential moderators. We delineated five subgroups: type of problematic media use (categorized as gaming, internet, smartphone, and social media), geographical region (categorized as Asia, Europe, and North America), lag length in months (categorized as lag ≤3, 3< lag ≤6, and lag >6), measure of problematic media use (categorized as validated, Diagnostic and Statistical Manual of Mental Disorders [DSM] criteria-based, and adapted measures), and measure of loneliness (categorized as validated and adapted measures). Although we recorded the specific measures of loneliness and problematic media use for each study, it is noteworthy that certain measures were infrequently used across studies (eg, Generalized Problematic Internet Use Scale [GPIUS]). Consequently, this scarcity hindered our ability to generate meaningful comparisons within these specific measures.

Finally, contour-enhanced funnel plots were generated to visually inspect for potential publication bias. Egger regression tests were conducted to provide a more robust statistical evaluation of funnel plot asymmetry. In addition, the correlation between sample size and effect size was examined, with a negative correlation indicating potential publication bias [33].

## Results

### Screening and Selection Process

**Figure 1.**
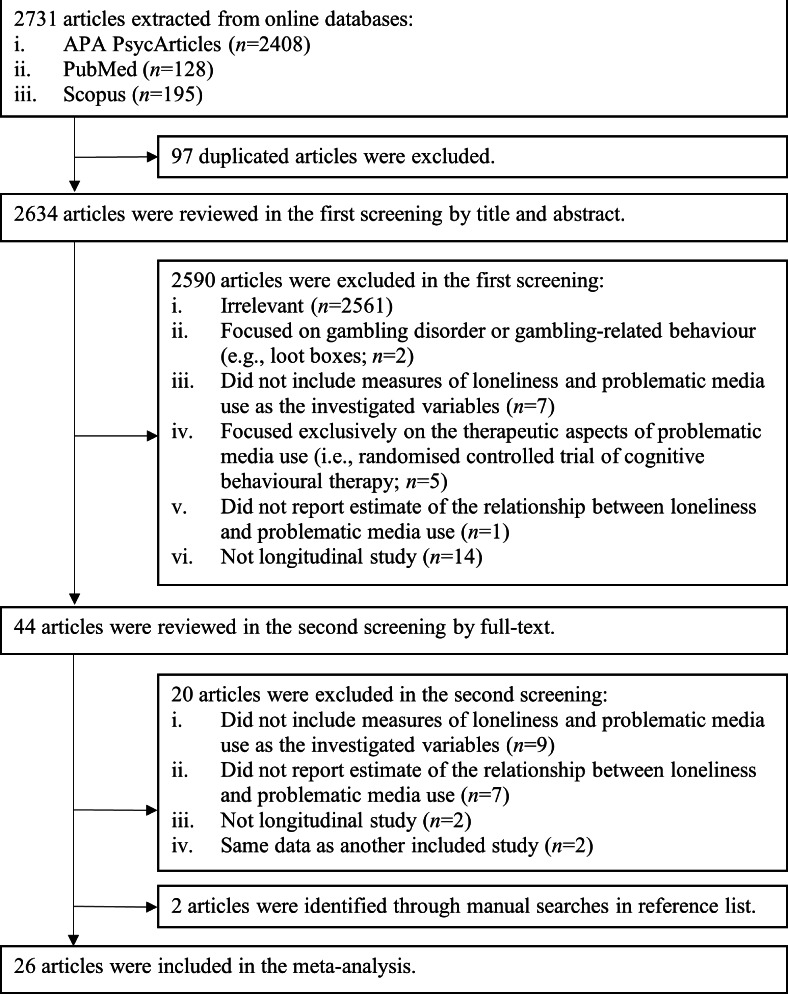
Flowchart of the data screening process.

A total of 2731 records were retrieved from 3 online databases (APA PsycArticles=2408, PubMed=128, and Scopus=195). After removing 97 duplicated papers, 2634 records remained and were screened based on titles and abstracts. Of these, 2590 papers were further excluded. The remaining 44 papers, along with key studies identified through manual searches in the reference list, were selected for full-text review. Finally, a total of 26 relevant studies were included for the meta-analysis (Figure 1).

### Descriptive Characteristics of the Selected

A summary of the included studies was presented in Table 1. The 26 studies involve a total of 24,798 individuals from diverse backgrounds, ranging from adolescents [34] to smartphone users in the general population [18].

**Table 1.** Summary of studies included in the meta-analysis.

Study and reference	Country (region)	Population	Sample size (n)	Media	PMU^a^ measure	Loneliness measure
Fang et al [25]	China (Asia)	Middle school students	677	Internet	PIUS^b^	UCLA 3^c^
Finserås et al [35]	Norway (Europe)	Adolescents	1258	Gaming	*DSM-5* IGD^d^	RULS-8^e^
Gao et al [14]	China (Asia)	Undergraduates	367	Gaming	OGAS^f^	ULS-8^g^
Gao et al [36]	China (Asia)	Freshman	319	Gaming	OGAS	ULS-8
Hu and Xiang [34]	China (Asia)	Adolescents	906	Smartphone	Adapted	Adapted
Jia et al [37]	China (Asia)	First-year university students	361	Internet	IAT^h^	UCLA^i^
Karsay et al [18]	Germany (Europe)	Smartphone users	461	Smartphone	Adapted	Adapted
Kim [16]	Korea (Asia)	Smartphone users	288	Smartphone	Adapted	UCLA 3
Krossbakken et al [38]	Norway (Europe)	Adolescents	1277	Gaming	GAS^j^	RULS-8
Lapierre et al [21]	United States (North America)	Late adolescents	346	Smartphone	SPS^k^	ULS-8
Lemmens et al [39]	Netherlands (Europe)	Game-playing adolescents	543	Gaming	GAS	UCLA 3
Mun [40]	Korea (Asia)	Adolescents	393	Gaming	Adapted	Adapted
Ok [41]	Korea (Asia)	Students	529	Gaming	IAT	UCLA
Olsen et al [42]	Norway (Europe)	Individuals in military service	258	Gaming	GAS	RULS-8
Reed et al - study 2 [22]	United Kingdom (Europe)	University students	32	Internet	IAT	UCLA
Reed et al - study 2 [22]	United Kingdom (Europe)	University students	41	Internet	IAT	UCLA
Rogier et al [15]	Italy (Europe)	Adults	308	Gaming Social media	IGDS9-SF^l^ BSMAS^m^	UCLA
Shi [43]	China (Asia)	College students	3827	Smartphone	SAS-SV^n^	ULS-8^o^
Tian [44]	China (Asia)	University students	291	Internet	IAT	UCLA
Tian [45]	China (Asia)	Junior middle school students	1047	Internet	IAT	UCLA
Tóth‐Király [46]	Finland (Europe)	Adolescents	1736	Internet	Adapted	Adapted
Wang [47]	China (Asia)	University freshmen and sophomores	7434	Smartphone	SAS-SV	ULS-8
Wang [48]	China (Asia)	6th-grade students	296	Internet	GPIUS^p^	UCLA 3
Wu [49]	China (Asia)	University students	538	Social media	PMSMUAS^q^	UCLA 3
Yao [50]	United States (North America)	College freshmen	219	Social media	Adapted	SLS^r^
Yang [51]	Hong Kong (Asia)	College students	361	Internet	IAT	UCLA 3
Zhou [23]	China (Asia)	First-year undergraduates	685	Social media	BSMAS	ULS-6^s^

a PMU: problematic media use.

b PIUS: Pathological Internet Use Scale.

c UCLA 3: UCLA Loneliness Scale Version 3.

d *DSM-5* IGD: *Diagnostic and Statistical Manual of Mental Disorders, Fifth Edition,* IGD criteria

e RULS-8: Roberts UCLA Loneliness Scale.

f OGAS: Online Game Addiction Scale.

g ULS-8: Short-Form Loneliness Scale.

h IAT: Young’s Internet Addiction Test.

i UCLA: UCLA Loneliness Scale.

j GAS: Game Addiction Scale.

k SPS: Smartphone Proneness Scale.

l IGDS9-SF: Internet Gaming Disorder Scale–Short Form.

m BSMAS: Bergen Social Media Addiction Scale.

n SAS-SV: Smartphone Addiction Scale–Short Version.

o ULS-8: Short-Form Loneliness Scale.

p GPIUS: Generalized Problematic Internet Use Scale.

q PMSMUAS: Problematic Mobile Social Media Usage Assessment Scale for Adolescents.

r SLS: Short Loneliness Scale.

s ULS-6: 6-item Chinese version of UCLA Scale.

### Risk of Bias Assessment

The JBI Critical Appraisal Checklist for Cohort Studies was used to evaluate the risk of bias (Table 2). The maximum score among the studies was 9 (range 3‐9). The average quality score in the studies was approximately 7 points. In 23 studies, the methodological quality was at least 50% of the total score. Notably, a high risk of bias was observed in 3 studies [21,22,50]. A sensitivity analysis was performed by comparing datasets with and without these 3 studies (findings not reported here). The results of the sensitivity analysis were identical between the 2 datasets. Therefore, the 3 studies were retained in the analysis for better coverage.

**Table 2.** Summary of quality appraisal based on the Joanna Briggs Institute Critical Appraisal Checklist for Cohort Studies. The maximum score and appropriate appraisal for cohort studies were used with 11 criteria.

Study and reference	1	2	3	4	5	6	7	8	9	10	11	Total
Fang et al [25]	H^a^	L^b^	L	L	L	NA^c^	L	L	L	NA	L	8/9
Finserås et al [35]	L	L	L	L	L	NA	L	L	H	NA	L	8/9
Gao et al [14]	H	L	L	L	L	NA	L	H	H	NA	L	6/9
Gao et al [36]	H	L	L	L	L	NA	L	L	L	NA	L	8/9
Hu and Xiang [34]	H	L	L	H	H	NA	L	L	H	NA	L	5/9
Jia et al [37]	H	L	L	H	H	NA	L	L	L	NA	L	6/9
Karsay et al [18]	L	H	L	L	L	NA	L	L	L	NA	L	8/9
Kim [16]	H	L	L	L	L	NA	L	H	H	NA	L	6/9
Krossbakken et al [38]	L	L	L	L	L	NA	L	L	L	NA	L	9/9
Lapierre et al [21]	H	H	L	H	H	NA	L	H	H	NA	L	3/9
Lemmens et al [39]	H	L	L	L	L	NA	L	L	H	NA	L	7/9
Mun [40]	H	H	L	L	L	NA	L	L	L	NA	L	7/9
Ok [41]	H	H	L	L	L	NA	L	L	H	NA	L	6/9
Olsen et al [42]	H	L	L	H	H	NA	L	L	L	NA	L	6/9
Reed et al - study 1 [22]	H	H	L	H	H	NA	L	L	L	NA	H	4/9
Reed et al - study 2 [22]	H	H	L	H	H	NA	L	L	L	NA	H	4/9
Rogier et al [15]	H	L	L	L	L	NA	L	H	L	NA	L	7/9
Shi et al [43]	H	L	L	L	L	NA	L	L	H	NA	L	7/9
Tian et al [44]	H	L	L	H	H	NA	L	L	L	NA	L	6/9
Tian et al [45]	H	L	L	L	L	NA	L	L	L	NA	L	8/9
Tóth‐Király et al [46]	L	L	L	L	L	NA	L	L	L	NA	L	9/9
Wang et al [47]	L	L	L	L	L	NA	L	L	L	NA	L	9/9
Wang et al [48]	L	L	L	L	L	NA	L	L	H	NA	L	8/9
Wu et al [49]	H	L	L	H	L	NA	L	L	L	NA	L	7/9
Yao and Zhong [50]	H	H	L	H	H	NA	L	H	H	NA	L	3/9
Yang et al [51]	L	L	L	L	L	NA	H	L	L	NA	L	8/9
Zhou et al [23]	L	L	L	H	H	NA	L	L	L	NA	L	7/9

a H: high risk of bias (scored as 0).

b L: low risk of bias (scored as 1).

c NA: not applicable (excluded from the total score).

### Longitudinal Relationship From Loneliness to Problematic Media Use

Based on the random-effects model, a significant relationship was observed between loneliness at Time 1 and problematic media use at Time 2 (refer to Table 3). Specifically, the strength of this relationship was notably higher when examined using the Pearson correlation coefficient (*r*=0.28, 95% CI 0.25-0.31; prediction interval 0.11-0.44) compared with other research models (*r*=0.10, 95% CI 0.08-0.13; prediction interval −0.02 to 0.23) and estimated beta coefficients (*r*=0.10, 95% CI 0.09-0.12; prediction interval 0.05-0.16).

**Table 3.** Longitudinal relationship from loneliness to problematic media use.

Moderator	Pearson						Model						Estimated					
	*k^a^*	*r* (95% CI)	*τ* ^2^	*I* ^2^	Q value	*P* value	*k*	*r* (95% CI)	*τ* ^2^	*I* ^2^	Q value	*P* value	*k*	*r* (95% CI)	*τ* ^2^	*I* ^2^	Q value	*P* value
Overall	33	0.28 (0.25-0.31)	0.09	86.1	105.59	<.001	32	0.10 (0.08-0.13)	0.00	68.8	105.59	<.001	28	0.10 (0.09-0.12)	0.00	38.6	105.59	<.001
Type of PMU^b^					28.55	<.001					0.55	.91					16.58	<.001
Social media	6	0.26 (0.18-0.33)	0.01	78.4			5	0.12 (0.03-0.22)	0.01	82.7			5	0.09 (0.03-0.15)	0.00	61.0		
Smartphone	8	0.36 (0.33-0.40)	0.00	81.5			5	0.09 (0.07-0.11)	0.00	0.0			8	0.13 (0.12-0.14)	0.00	0.0		
Internet	11	0.24 (0.21-0.26)	0.00	0.0			11	0.09 (0.06-0.13)	0.00	62.0			11	0.08 (0.05-0.10)	0.00	0.0		
Gaming	8	0.26 (0.19-0.33)	0.01	75.2			11	0.11 (0.05-0.16)	0.01	78.5			4	0.09 (0.04-0.14)	0.00	0.0		
Country					7.69	.02					0.64	.73					0.36	.83
Asia	27	0.30 (0.27-0.33)	0.01	85.5			19	0.11 (0.07-0.14)	0.00	67.7			24	0.10 (0.08-0.12)	0.00	45.8		
Europe	4	0.20 (0.12-0.28)	0.00	54.9			12	0.10 (0.06-0.15)	0.00	73.5			2	0.11 (0.04- 0.18)	0.00	0.0		
North America	2	0.19 (0.06-0.31)	0.01	58.5			1	0.06 (−0.05 to 0.16)	—	—			2	0.08 (−0.00 to 0.16)	0.00	0.0		
Lag length (month)					2.76	.25					7.60	.02					0.66	.72
Lag ≤3	6	0.26 (0.22-0.30)	0.00	0.0			8	0.18 (0.12-0.24)	0.00	57.6			3	0.08 (0.02-0.14)	0.00	0.0		
3< lag ≤6	15	0.31 (0.26-0.36)	0.01	87.9			10	0.08 (0.05-0.12)	0.00	57.1			13	0.10 (0.08-0.13)	0.00	61.1		
Lag >6	12	0.26 (0.21-0.31)	0.01	72.8			14	0.09 (0.05-0.12)	0.00	72.0			12	0.10 (0.07-0.12)	0.00	0.0		
Measure of PMU					0.76	.38					8.15	.02					0.08	.78
Validated	28	0.28 (0.25-0.31)	0.01	86.9			26	0.10 (0.07-0.12)	0.00	64.9			23	0.10 (0.08-0.12)	0.00	46.2		
DSM^c^ criteria	—	—	—	—			1	0.02 (–0.04 to 0.07)	—	—			—	—	—	—		
Adapted	5	0.33 (0.21-0.44)	0.02	84.1			5	0.14 (0.06-0.22)	0.01	78.0			5	0.10 (0.06-0.14)	0.00	0.0		
Measure of loneliness					2.50	.11					1.25	.26					0.13	.72
Validated	30	0.28 (0.25-0.31)	0.01	87.3			28	0.09 (0.07-0.12)	0.00	64.8			25	0.10 (0.09-0.12)	0.00	41.5		
Adapted	3	0.33 (0.28-0.37)	0.00	10.7			4	0.15 (0.05-0.24)	0.01	83.4			3	0.09 (0.05-0.14)	0.00	0.0		

a *k*: number of estimates.

b PMU: problematic media use.

c *DSM*: *Diagnostic and Statistical Manual of Mental Disorders.*

### Longitudinal Relationship From Problematic Media Use to Loneliness

A significant relationship was observed between problematic media use at Time 1 and loneliness at Time 2 (Table 4). Similar to the other direction, the relationship is significantly stronger when examined using the Pearson correlation coefficient (*r*=0.29, 95% CI 0.26-0.32; prediction interval 0.13-0.43) compared with other research models (*r*=0.13, 95% CI 0.07-0.13; prediction interval −0.02 to 0.23) and estimated beta coefficients (*r*=0.09, 95% CI 0.07-0.11; prediction interval 0.01-0.16).

**Table 4.** Longitudinal relationship from problematic media use to loneliness.

Moderator	Pearson						Model						Estimated					
	*k^a^*	*r* (95% CI)	*τ* ^2^	*I* ^2^	Q value	*P* value	*k*	*r* (95% CI)	*τ* ^2^	*I* ^2^	Q value	*P* value	*k*	*r* (95% CI)	*τ* ^2^	*I* ^2^	Q value	*P* value
Overall	27	0.29 (0.26-0.32)	0.01	85.2	110.13	<.001	23	0.10 (0.07-0.13)	0.00	73.3	110.13	<.001	27	0.09 (0.07-0.11)	0.00	52.9	110.13	<.001
Type of PMU^b^					4.18	.24					3.75	.29					1.86	.60
Social media	4	0.32 (0.21-0.41)	0.01	87.2			4	0.08 (0.03-0.12)	0.00	0.0			4	0.08 (0.04-0.12)	0.00	0.0		
Smartphone	9	0.31 (0.27-0.36)	0.00	86.7			5	0.09 (0.03-0.15)	0.00	70.9			9	0.08 (0.07-0.09)	0.00	9.9		
Internet	11	0.25 (0.20-0.29)	0.00	65.8			9	0.23 (0.07-0.39)	0.06	86.8			11	0.12 (0.06-0.18)	0.01	75.7		
Gaming	3	0.30 (0.18-0.41)	0.01	82.8			5	0.07 (0.05- 0.10)	0.00	0.0			3	0.10 (0.05-0.15)	0.00	0.0		
Country					10.70	.00					2.05	.36					0.09	.95
Asia	24	0.30 (0.26-0.33)	0.01	85.0			14	0.09 (0.06-0.12)	.00	61.4			24	0.09 (0.07-0.11)	0.00	57.6		
Europe	2	0.19 (0.12-0.24)	0.00	0.0			8	0.23 (0.04-0.41)	.08	85.5			2	0.08 (0.02-0.14)	0.00	0.0		
North America	1	0.25 (0.15-0.35)	—	—			1	0.08 (–0.03 to 0.18)	—	—			1	0.08 (–0.03 to 0.18)	—	—		
Lag length (month)					1.13	.57					9.74	.01					1.26	.53
Lag ≤3	3	0.27 (0.15-0.38)	0.01	76.6			4	0.40 (0.02-0.68)	0.00	62.1			3	0.14 (–0.02 to 0.29)	0.02	*85.5*		
3< lag ≤6	13	0.30 (0.26-0.35)	0.01	85.2			9	0.12 (0.08-0.17)	0.00	62.1			13	0.09 (0.07-0.10)	0.00	*52.4*		
Lag >6	11	0.27 (0.22-0.32)	0.01	80.0			10	0.16 (0.04-0.08)	0.00	35.7			11	0.07 (0.04-0.10)	0.00	*14.8*		
Measure of PMU					0.05	.83					0.02	.88					3.27	.07
Validated	22	0.29 (0.25-0.32)	0.01	86.2			21	0.10 (0.07-0.12)	0.00	70.9			22	0.10 (0.07-0.12)	0.00	58.1		
Adapted	5	0.30 (0.21-0.37)	0.01	78.1			2	0.11 (–0.09 to 0.31)	0.02	92.6			5	0.05 (0.01-0.09)	0.00	0.0		
Measure of loneliness					.08	.78					0.02	.88					2.15	.14
Validated	23	0.29 (0.25-0.32)	0.01	85.6			21	0.10 (0.07-0.12)	0.00	70.9			23	0.09 (0.07-0.12)	0.00	57.3		
Adapted	4	0.28 (0.19-0.36)	0.01	78.3			2	0.11 (–0.09 to 0.31)	0.02	92.6			4	0.06 (0.02-0.10)	0.00	0.0		

a k: number of estimates.

b PMU: problematic media use.

### Subgroup Analysis

When examining the relationship using Pearson correlation coefficients, we found that country acted as a significant moderator for the longitudinal relationship in both directions, particularly showing stronger relationships in the Asian region (loneliness → problematic media use: *Q*=7.69; *P*=.02 and problematic media use → loneliness: *Q*=10.70; *P*=.01). On the other hand, the type of problematic media use also acted as a significant moderator in the longitudinal relationship from loneliness to problematic media use, with a stronger relationship in the case of smartphone addiction (*Q*=28.5; *P*<.001).

In contrast, the majority of subgroup analyses based on research models and estimated beta coefficients did not yield significant results, suggesting a high level of stability in the findings across the 2 methods. While problematic media use emerged as a significant moderator when examining the relationship with beta coefficients from various statistical models, it is important to note that only 1 study used DSM criteria to measure problematic media use [35]. Exploratory data analysis with the exclusion of this study revealed a nonsignificant moderating effect with regard to the measure of problematic media use (finding is not shown in this paper). Overall, the subgroup analyses provide general support for the bidirectional relationship between loneliness and problematic media use.

### Publication Bias

**Figure 2.**
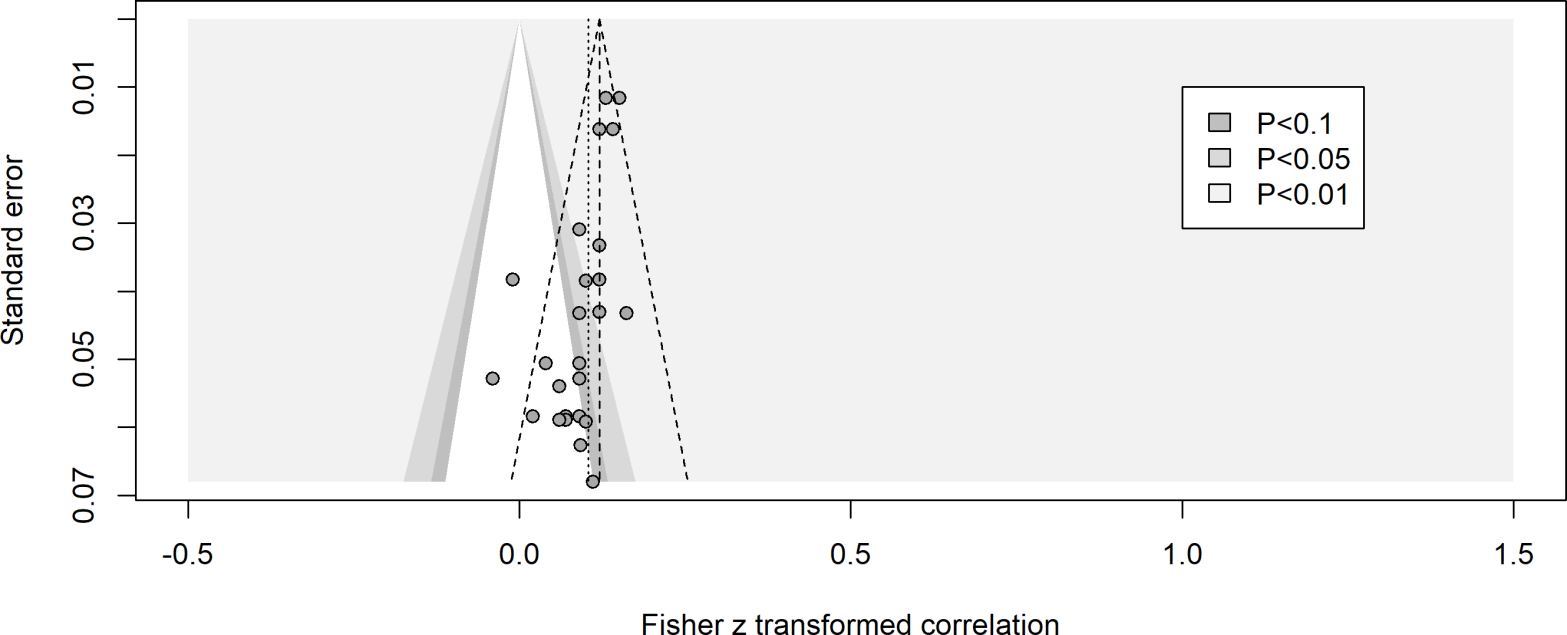
Funnel plot for studies examining the longitudinal relationship from loneliness to problematic media use.

Contour-enhanced funnel plots and Egger regression test were performed separately for the 2 causal directions to identify potential publication bias. The funnel plot for the direction of loneliness to problematic media use was asymmetrical (Figure 2), with a bias estimate of −1.55, *t*_26_=−4.94; *P*<.001. It is noteworthy that studies with lower SEs scatter in the middle of the funnel plot, while studies with larger SEs generally scatter around the left side of the funnel plot. Additional analysis revealed a nonsignificant relationship between sample size and effect size, *r*(91)=0.135, 95% CI −0.070 to 0.330; *P*=.20.

**Figure 3.**
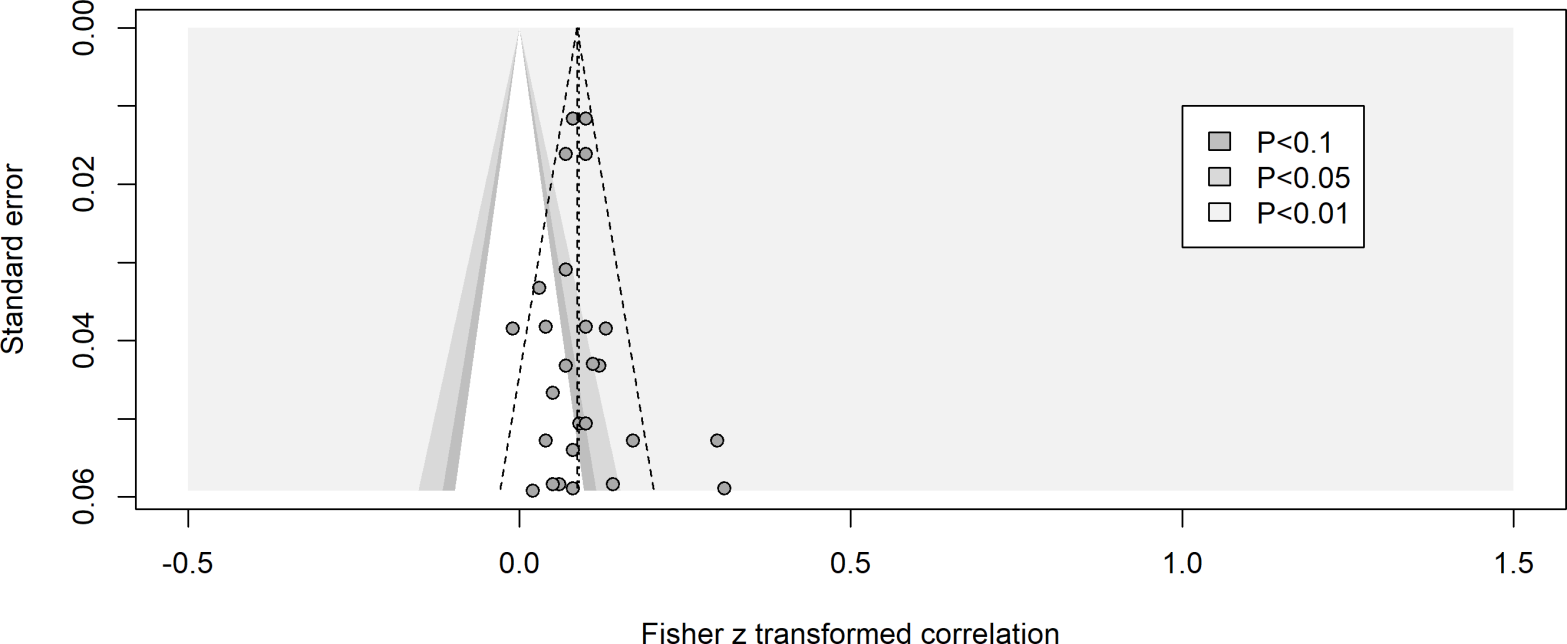
Funnel plot for studies examining the longitudinal relationship from problematic media use to loneliness.

On the other hand, the funnel plot for the direction of problematic media use to loneliness is symmetrical (Figure 3), with a bias estimate of 0.32, *t*_25_=0.62; *P*=.54. Additional analysis revealed a nonsignificant relationship between sample size and effect size, with *r*(75)=−0.004, 95% CI −0.228 to 0.220; *P*=.97). These results generally suggest a low risk of publication bias.

## Discussion

### Principal Findings

Using a random-effects model, the current meta-analysis synthesized the longitudinal relationships between loneliness and problematic media use. Given the contrasting perspectives on the direction of causality, 2 separate analyses were carried out for both directions. Three types of effect sizes were extracted from 26 longitudinal studies: Pearson correlation coefficients, standardized beta coefficients from various statistical models, and estimated beta coefficients based on Cohen et al’s [28] formula.

### Longitudinal Relationships Between Loneliness and Problematic Media Use

Regardless of the type of effect size, the results consistently revealed significant longitudinal relationships between loneliness and problematic media use in both directions. The findings support a bidirectional model where loneliness and problematic media use can influence each other over time. Consistent with the compensatory internet use theory [17], lonely individuals may initially use the internet to fulfill unmet social needs, finding temporary relief through online connection. These individuals might become increasingly dependent on the internet to fulfill their social needs, resulting in problematic media use [52]. Conversely, as highlighted in the internet paradox [5], excessive internet use can displace real-world interactions and weaken social skills. In addition, the quality of online interactions may not fully satisfy social needs, leading to a sense of loneliness despite being connected online.

### Explaining Heterogeneity

This meta-analysis revealed a medium effect size when examining longitudinal relationships with Pearson correlation coefficients ($r_{LPIU}$=0.28; $r_{PIUL}$ =0.29). This finding aligns with a previous meta-analysis by Zhang et al ($r_{LPIU}$=029; $r_{PIUL}$ =0.26) [24]. However, the effect sizes were weaker when the relationships were examined using standardized beta coefficients (β=.10; β=.10) or estimated beta coefficients (β=.10; β =.09). This suggests that beta coefficients may provide a more accurate estimate of the longitudinal relationships between loneliness and problematic media use (with narrower CIs), particularly by accounting for the influence of the outcome variable at Time 1.

Furthermore, both types of beta coefficients exhibit greater stability across diverse study designs compared with Pearson correlation coefficients (eg, geographical region, type of measures, and lag length). Based on these findings, it is recommended for future meta-analyses examining longitudinal relationships to prioritize the use of beta coefficients obtained from robust research models such as cross-lagged models. In addition, individual studies are recommended to report a complete correlation matrix across all variables and time points. These practices would enhance the accuracy and interpretability of findings on longitudinal relationships.

The estimated 95% prediction intervals indicate that future studies using Pearson correlation coefficients are likely to consistently yield positive longitudinal relationships between loneliness and problematic media use. In contrast, studies using standardized beta coefficients or estimated beta coefficients may produce more variable findings, including potential null or negative relationships. Given the moderate to high heterogeneity reflected by the *I*^2^ statistic, it is plausible that covariates may influence the observed effect sizes. This study highlights the need for additional longitudinal research from diverse contexts to enhance the robustness of the evidence base and clarify the factors contributing to these relationships. Notably, with approximately 60% of the included studies conducted in Asia, fostering greater international collaboration would provide a more comprehensive understanding and improve generalizability across populations.

The current findings suggest weaker longitudinal relationships between loneliness and problematic media use compared with those reported by the previous meta-analysis [24]. While methodological differences might be the primary cause for the discrepancy, the findings could also be partially explained by the evolving nature of the internet. Kraut et al [53] revisited the internet paradox and found a more positive long-term impact a few years after their initial study, attributing it to technology advancements that facilitated healthier online social interactions. Nearly 2 decades after the study [53], the internet offers a wider array of social functions through social media, smartphones, and online gaming. These advancements may have indeed fostered more positive social interaction experiences within online environments [54]. For instance, instant messaging allows connection with offline friends, potentially facilitating discussions of more intimate topics [55]. Similarly, online and offline gaming communities can connect individuals with similar interests, promoting a sense of belonging. Therefore, the maturing and diverse functionalities of the internet may contribute to healthy friendship formation and maintenance, potentially weakening the longitudinal relationships between loneliness and problematic media use. Future studies could explore specific aspects of the internet (eg, social media features) that foster healthier social interactions.

Finally, it is important to acknowledge that the current meta-analysis focused on problematic media use (ie, internet gaming disorder, social media disorder, internet addiction, or smartphone addiction). The magnitude of the longitudinal relationships might be stronger if the focus is on general media use. On one hand, loneliness might lead individuals to engage more in online social interactions, but not to the extent of problematic use [56]. On the other hand, spending long hours on the internet might result in reduced social interactions and contribute to feelings of loneliness [5,57]. Further complicating the relationship, some studies suggest that internet use can effectively decrease loneliness [58]. This highlights the importance of considering the different purposes of internet use beyond solely focusing on symptoms of behavioral addiction. For example, one longitudinal study [59] found that the social use of the internet, specifically online interactions with friends and family, can decrease loneliness and increase social engagement. In contrast, informational and instrumental uses, such as browsing news or searching for information, were not related to loneliness. These findings suggest that future research should examine the longitudinal relationships between loneliness and general media use or internet use and explore the potential moderating role of different purposes of internet use.

### Limitations and Future Directions

This meta-analysis has several limitations. First, although the contour-enhanced funnel plot and Egger regression test suggest an asymmetrical pattern in studies examining the direction of loneliness to problematic media use, it is notable that the studies seem to be missing in the area of significance (*P*<.01). Hence, the asymmetrical pattern might not be the outcome of publication bias or selective nonreporting of results [60]. One possible explanation for this outcome is between-study heterogeneity (*I*^2^=38.6%); however, more longitudinal studies are needed to confirm this.

Second, a few studies reported nonsignificant longitudinal relationships without specifying the actual coefficients [37,43,44]. With the nonsignificant findings primarily found in the cross-lagged model, the actual relationships might be even smaller than the current findings.

Third, it is important to note that moderate to high heterogeneity was observed in several subgroups. This suggests the presence of additional covariates that were not accounted for in this study. For instance, one of the included studies [46] proposed that both social and individual antecedents could influence the relationship, highlighting the need for future research to explore these factors in greater depth.

Fourth, there are a few studies that met the inclusion criteria but did not report the estimate of the hypothesized longitudinal relationships (most of these studies focused on other relationships). While attempts were made to contact authors for missing data, most did not respond. Finally, the screening process was limited to studies published exclusively in English, potentially excluding valuable information from other linguistic sources.

### Conclusion

In conclusion, the findings support a bidirectional model, suggesting that loneliness and problematic media use can influence each other over time. While significant relationships were found in both directions, the effect sizes were weaker than anticipated. This may be partially explained by the evolving nature of the internet, as evidenced by advancements that facilitate healthier online social interactions. In addition, subgroup analyses revealed greater stability in the relationships when examined using beta coefficients, which account for previous outcome variables.

## Supplementary material

10.2196/60410 Checklist 1 PRISMA (Preferred Reporting Items for Systematic Reviews and Meta-Analyses) 2020 abstract checklist.

## Abbreviations

*DSM-5*: *Diagnostic and Statistical Manual for Mental Disorders, Fifth Edition*
DV: dependent variable
GPIUS: Generalized Problematic Internet Use Scale
JBI: Joanna Briggs Institute
OSF: Open Science Framework
PRISMA: Preferred Reporting Items for Systematic Reviews and Meta-Analyses
